# Association of physical activity on memory interference: Boston Puerto Rican Health Study

**DOI:** 10.34172/hpp.2021.31

**Published:** 2021-05-19

**Authors:** Paul D. Loprinzi, Lindsay K. Crawford, Tammy Scott, Katherine L. Tucker

**Affiliations:** ^1^Exercise & Memory Laboratory, Department of Health, Exercise Science and Recreation Management, University of Mississippi, Mississippi, USA; ^2^Friedman School of Nutrition Science and Policy, Tufts University School of Medicine, Boston, MA, USA; ^3^Center for Population Health, Biomedical & Nutritional Sciences, University of Massachusetts Lowell, Lowell, Ma, USA

**Keywords:** Cognition, Exercise, Memory

## Abstract

**Background** : The objective of this study was to evaluate the association between habitual physical activity engagement on memory interference. The present analysis used cross-sectional data from the Boston Puerto Rican Health Study (n=1,241; mean age= 57.2; 72.1% female).

**Methods** : Physical activity was evaluated via self-report. Memory interference was evaluated using a word-list paradigm. The memory task included learning a list of 16 words (List A; 5 trials), followed by a distractor list (List B), and then an immediate recall of List A. Proactive interference occurs when preceding stimuli (e.g., Trial 1 and Trial 5 of List A) interferes with performance on a subsequent stimuli (List B). Retroactive interference occurs when subsequent stimuli (List B) interferes with the recall of previously encoded stimuli (Trial 5).

**Results** : For proactive interference, there was no association between physical activity and the difference between performance on List B and Trial 1 of List A (β=0.00001; *P* =0.96). Similarly, for retroactive interference, there was no association between physical activity and the difference between the short delay recall and Trial 5 of List A (β=0.0002; P=0.50).

**Conclusion** : The present study did not observe an association between habitual physical activity on attenuating memory interference.

## Introduction

The health-enhancing cardiometabolic benefits of physical activity are well-established.^1,2^ Accumulating research has also demonstrated neuroprotective effects of physical activity on the brain.^3,4^ For example, research demonstrates that both acute^5-9^ and chronic^3,4,10^ physical activity may enhance memory function and attenuate age-associated decline in memory.

Of interest to this brief report is whether habitual physical activity can attenuate a potential memory interference effect. There are two main types of memory interference, namely proactive interference and retroactive interference. Proactive interference occurs when preceding stimuli interferes with performance on a subsequent stimuli. On the other hand, retroactive interference occurs when subsequently encoded stimuli interferes with the recall of previously encoded stimuli.

Not only can acute and chronic physical activity enhance memory function,^3-10^ there is plausibility for physical activity to minimize a memory interference effect. For example, Guise and Shapiro demonstrated, within an animal model, that the prefrontal cortex plays an important role in minimizing proactive memory interference.^11^ Specifically, they showed that when the medial prefrontal cortex is inhibited, hippocampal encoding is impaired, subsequently preventing learning ability during interfering stimuli. Thus, these findings suggest that the prefrontal cortex and hippocampus work together to retrieve distinct information from similar contextual stimuli, which should assist in the attenuation of memory interference. Notably, research has shown that habitual physical activity is associated with increased prefrontal cortex and hippocampal volume,^12^ and also is associated with increase prefrontal cortex activity during cognitive tasks that require concurrent processing of multiple stimuli.^13,14^ Further, physical activity may subserve hippocampal-dependent memory function via physical activity-induced alterations in key neurotransmitters (e.g., dopamine, serotonin, acetylcholine, norepinephrine, and brain-derived neurotrophic factor) that facilitate long-term potentiation.^10^

Our group has experimentally evaluated the effects of acute exercise on both proactive and retroactive interference.^15-17^ In these experiments,^15-17^ we provide suggestive evidence that acute exercise may help to minimize a memory interference effect, specifically retroactive interference. However, these associations failed to reach statistical significance, which may have been a result of low statistical power due to small sample sizes. As an extension of this work, within this novel line of inquiry, the main objective of the present paper was to examine the relationship between habitual physical activity on memory interference in a large sample of adults.

## Materials and Methods

### Study design and participants

Data from the Boston Puerto Rican Health Study was utilized, with details of this federally funded (NIH) study described elsewhere.^18^ Ethical approval (#6763) was obtained and all participants provided written consent. The sample consisted of Puerto Rican adults, aged 45-75 years, residing in Boston, Massachusetts, USA. In totality, 2170 individuals were identified, and among these, 1811 agreed to participate. Of these 2170 individuals, 1500 individuals participated in the initial interview. For the present paper, the sample included 1241 participants, whom had complete data. Inclusionary criteria included self-identified Puerto Rican decent, aged 45-75 y, able to answer questions in English or Spanish, and living in the Boston, MA metropolitan area. Exclusionary criteria included an inability to answer questions due to serious health conditions, plans to move away from the area within two years, and a Mini Mental State Examination score ≤ 10.

### Measurement of physical activity

As reported previously,^18^ and evaluated among this Puerto Rican population,^19^ physical activity was evaluated via self-report methodology, modeled after the Paffenbarger survey.^20,21^ From estimates of sleep, light, moderate, and vigorous physical activity, daily expenditure was calculated based on the degree of oxygen consumption associated with each individual activity.^18,22^

### Measurement of memory

**Figure 1:**
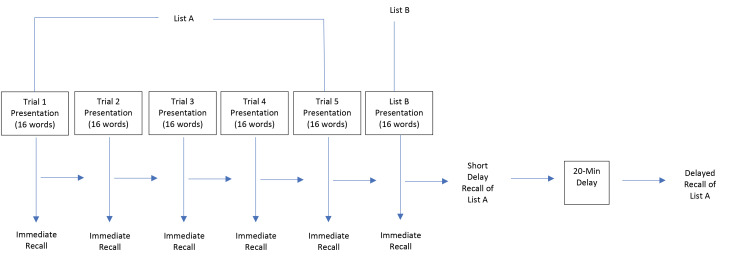
Schematic of the memory assessment protocol

As described elsewhere,^23,24^ memory function was administered during the baseline household interview. For the present study, a *List Learning* (episodic memory) protocol was employed. The List Learning task, which was developed as part of the California Verbal Learning Test, includes learning a list of 16 words (List A; 5 trials), followed by a distractor list (List B), and then a short delay recall of List A (Figure 1). The List A (e.g., giraffe, leg, bed) and List B (e.g., elephant, foot, dress) words were similar in content, which included nouns ranging from one to four syllables and three to 12 letters. Notably, similar list-learning tests have demonstrated adequate test-retest reliability.^25^

Proactive interference occurs when preceding stimuli (e.g., learning of List A) interferes with learning of a subsequent stimuli (List B). In this analysis, a comparison of recalls for Trial 1 and List B was used as a marker for proactive interference (List B – Trial 1).

Retroactive interference occurs when learning of a new stimuli (List B) interferes with the recall of previously encoded stimuli (short delay recall of List A). Thus, retroactive interference was assessed by comparison of short delay recall and recall of Trial 5 for List A (short delay recall – Trial 5).

### Statistical analyses 

To evaluate the association between physical activity and memory interference, multivariable linear regression analyses were computed, via Stata (v. 12, College Station, TX, USA). In all models, physical activity served as the independent variable.

To evaluate proactive memory interference, we examined the relationship between physical activity on the difference between List B and Trial 1 (List B - Trial 1).

To evaluate retroactive memory interference, we examined the relationship between physical activity on the difference between short delay recall and Trial 5 (short delay recall - Trial 5).

In these two models, covariates included sex, education, age, smoking, income, body mass index (BMI), perceived health status, literacy, depression, hypertension, diet (HEI-2005), diabetes and alcohol use. Statistical significance was set at an a-priori nominal alpha of 0.05.

## Results

Participants, on average, were 57.2 years old, with the majority of the sample being female (72.1%) (Table 1). The sample, on average, had a BMI in the obesity classification (31.8 kg/m^2^).

From Trials 1 to 5, there was a linear increase in the number of words recalled: 4.46 (1.7), 6.71 (2.2), 8.00 (2.6), 8.85 (2.8), and 9.55 (2.9) (Table 2). Following Trial 5, there was a reduced number of words recalled for List B: 3.70 (1.7). Lastly, following List B recall, participants recalled 7.57 (3.0) words for short delay recall of List A.

There was no evidence of an association between habitual physical activity and minimizing a memory interference effect (Table 3). For example, and for proactive interference, there was no association between physical activity and the difference between performance on List B and Trial 1 of List A (β=0.00001; *P* = 0.96). Similarly, for retroactive interference, there was no association between physical activity and the difference between the short delay recall and Trial 5 of List A (β=0.0002; *P* = 0.50).

## Discussion

Memory plays a critical role in daily functioning and emerging research demonstrates that both acute and habitual physical activity may help to enhance and preserve memory function.^3-10^ One factor that may influence the degree of retained information is whether interfering stimuli, of a similar context, occurs around the time of memory encoding. Such a memory interference effect appears to be largely influenced by prefrontal cortex functioning.^11^ Importantly, research demonstrates that both acute and chronic physical activity may help to enhance prefrontal cortex functioning,^12-14^ as well as enhance overall memory function.^3-10^ Recent experimental work, in predominately small samples, has started to evaluate whether physical activity can attenuate a memory interference effect.^15-17^ Among Puerto Rican middle-age to older adults, the present analysis evaluated the association between habitual physical activity and memory interference. Our main finding was that habitual physical activity was not associated with memory interference.

There are several possible explanations for our observed findings. It is possible that habitual physical activity is not associated with attenuating a memory interference effect. This aligns with recent experimental work failing to conclusively show that acute physical activity can attenuate a memory interference effect.^15-17^ An alternative possibility is that our observed null associations may be a result of the methodological nature of the present study. Most notably, physical activity was measured via self-reported methodology. Research demonstrates that subjective assessment of physical activity, when compared to objective measures of physical activity (e.g., accelerometry), attenuates associations toward the null, increasing the likelihood of committing a type II research error.^26^ Another possibility is that there was not sufficient variation in physical activity in this sample, as most participants were sedentary.

In conclusion, the present study did not observe an association between habitual physical activity on attenuating memory interference. Despite the notable strengths of this study, which includes, for example, the study novelty and large sample, future research should aim to overcome some of the limitations of the present study. As noted, future work on this novel line of inquiry would benefit by employing an objective measure of physical activity. Given the beneficial effects of acute physical activity on memory, particularly the temporal effects of acute physical activity on memory, future work that evaluates the association of habitual physical activity on memory interference should aim to disentangle any potential synergistic or inhibitory effects of acute and chronic exercise on memory interference (e.g., evaluating whether chronic physical activity is associated with memory interference when an acute bout of physical activity does not occur shortly before the memory task). Further, our sample was limited to Puerto Rican adults in the Boston, Massachusetts (USA) area. This population, compared to other populations, tends to have relatively high prevalence rates of conditions (depression,^27-29^ diabetes^30^ and obesity^31^) that may influence memory function. Thus, future work should evaluate this inquiry among other broader populations, including older adults.

## Funding

The present study was supported by the National Institute on Aging of the National Institutes of Health (NIH) (no. P01AG023394 and R01AG02708), the National Heart Lung and Blood Institute of NIH (no. P50HL105185) and the US Department of Agriculture, Agricultural Research Service contract (no. 58–1950–7–707).

## Competing interests

We have no conflicts of interest.

## Ethical approval

This project was approved by Tufts University IRB (#6763).

## Authors’ contributions

Author PL computed the analyses and prepared the initial draft of the manuscript. Authors LC, TS and KT wrote sections of the manuscript and provided feedback on multiple drafts of the manuscript. Author TS and KT were involved in designing the Boston Puerto Rican Health Study.

**Table 1:** Demographic and behavioral characteristics of the sample (n=1241)

**Variable**	**Point estimate**	**SD**
Age, mean years	57.2	7.5
Sex, % female	72.1	
Education, %		
No schooling or < 5^th^ grade	21.8	
5^th^ to 8^th^ grade	25.5	
9^th^ to 12^th^ grade	37.4	
Some college or bachelor’s degree	13.3	
At least some graduate school	1.8	
Income, mean annual household ($)	17 880	198 523
Body mass index, mean kg/m^2^	31.8	6.4
Hypertension (>140/90 mmHg), %	41.6	
Diabetes (glucose 126+ mg/dL or medication), %	39.7	
Depression Score, %		
15 or less	40.2	
16-21	15.6	
22+	44.2	
Literacy, % illiterate	7.6	
Health status, %		
Excellent	4.7	
Very good	6.0	
Good	18.6	
Fair	57.6	
Poor	13.1	
Diet, mean healthy eating index (HEI-2005)	71.5	9.6
Smoking, %		
Never	46.1	
Smoked in past, but not currently	30.0	
Current smoker	23.9	
Alcohol Use, %		
Never	28.3	
In the past, but not within past year	28.0	
Within past 30 days	26.7	
Within past year	17.0	
Physical activity score, mean	2148	379

**Table 2:** Memory function scores (n=1241)

**List learning**	**Point estimate (SD)**
List A, Trial 1, mean	4.46 (1.7)
Trial 2, mean	6.71 (2.2)
Trial 3, mean	8.00 (2.6)
Trial 4, mean	8.85 (2.8)
Trial 5, mean	9.55 (2.9)
List B, mean	3.70 (1.7)
List A, short delay recall, mean	7.57 (3.0)

**Table 3:** Multivariable linear regression analyses examining the association between physical activity (*independent variable*) and memory interference (n=1241)

**Physical activity**	**β**	**95% CI**	**P-Value**
	**Proactive Interference**		
PA on List B – List A Trial 1	0.00001	-0.0005, 0.0005	0.96
	**Retroactive Interference**		
PA on List A short delay recall – Trial 5	0.0002	-0.0003, 0.0008	0.50

Covariates included age, sex, education, income, BMI, hypertension, diabetes, depression, literacy, perceived health status, diet (HEI-2005), smoking, and alcohol use.
